# Endoscopic treatment of a large Brunner’s gland hamartoma in the duodenum

**DOI:** 10.1055/a-2336-3268

**Published:** 2024-06-26

**Authors:** Ha Eun Lee, Gwang Ha Kim, Kyungbin Kim

**Affiliations:** 1 58916 Department of Internal Medicine, Pusan National University School of Medicine, Busan, Korea (the Republic of); 2 220312 Biomedical Research Institute, Pusan National University Hospital, Busan, Korea (the Republic of); 3 220312 Department of Pathology, Pusan National University Hospital, Busan, Korea (the Republic of)

A 62-year-old woman visited our hospital for further evaluation of a large elongated polypoid mass in the duodenum, which was incidentally discovered during a health check-up endoscopy. The patient had a medical history of hypertension and hyperlipidemia. At endoscopy, a 10-cm subepithelial mass with a long stalk was observed in the second portion of the duodenum ( Fig. 1 **a** ); nodular mucosal changes were observed in the distal portion of the mass ( Fig. 1 **b** ). Endoscopic ultrasonography (EUS) revealed that the mass was a heterogeneously mixed echogenic lesion with cystic changes in the deep mucosal and submucosal layers Fig. 1 **c** ).

**Fig. 1:**
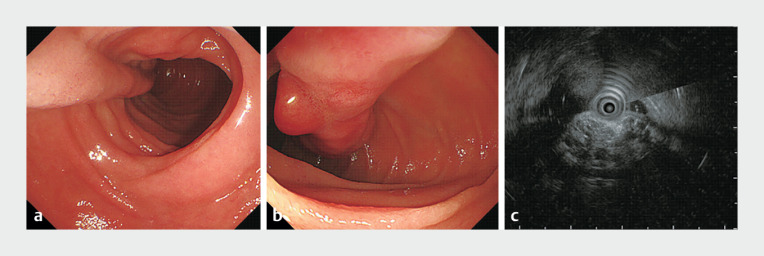
A duodenal subepithelial mass is seen on: **a, b** endoscopy; **c** endoscopic ultrasonography showing: **a** a 10-cm subepithelial mass with a long stalk in the second portion of the duodenum; **b** nodular mucosal changes in the distal portion of the mass; **c** a heterogeneously mixed echogenic mass with cystic changes in the deep mucosal and submucosal layers.

Endoscopic mucosal resection was planned to exclude the possibility of malignancy owing to the nodular mucosal changes on the surface of the mass and its large size ( Video 1 ). Because we were unable to place the snare at the base of the mass, it was first expelled from the duodenum into the stomach using mucosal forceps ( Fig. 2 **a, b** ). The snare was then placed at the base of the mass and the resection was performed ( Fig. 2 **c** ). Spurting bleeding occurred after the tumor resection ( Fig. 2 **d** ); however, hemostasis was successfully achieved by ligation using an O-ring ( Fig. 2 **e** ).

Endoscopic resection of a large Brunner’s gland hamartoma.Video 1

**Fig. 2:**
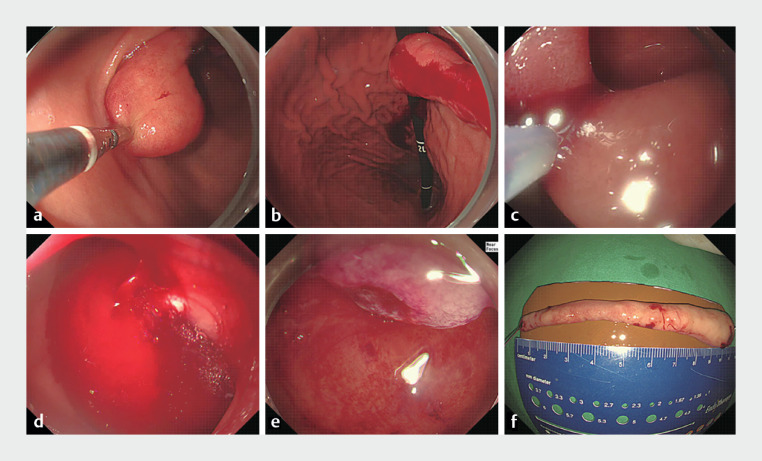
Endoscopic images of the resection of a duodenal subepithelial mass showing: **a, b** the mass being expelled from the duodenum to the stomach using mucosal forceps; **c** the snare being placed at the base of the mass, before resection is performed; **d** spurting bleeding after tumor resection; **e** successful hemostasis by ligation using an O-ring. **f** Photograph of the resected specimen, which was a 10.0 × 1.7-cm elongated subepithelial mass.

The resected specimen was a long polypoid subepithelial mass, measuring 10.0 × 1.7 cm ( Fig. 2 **f** ). Histopathologically, the mass comprised of proliferating adipose tissue and hyperplastic Brunner’s glands in the deep mucosa and submucosa, consistent with a Brunner’s gland hamartoma (BGH) ( Fig. 3 ). No dysplastic areas were observed in the resected tumor. The patient’s post-procedural course was uneventful and she was discharged on postoperative day 3.

**Fig. 3:**
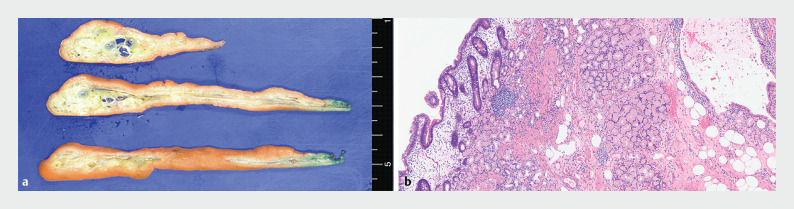
Histopathologic findings showing: **a** on gross evaluation, a well-defined heterogeneous yellow-to-white solid mass with cystic changes mainly located in the deep mucosa and submucosa; **b** microscopically, proliferating adipose tissue and hyperplastic Brunner’s glands, consistent with a Brunner’s gland hamartoma (hematoxylin and eosin [H&E] stain, magnification × 100).

BGH is a benign proliferative disorder of the Brunner’s glands in the duodenum 1 . It is often discovered incidentally during endoscopy, with typical findings indicating mucosal protrusions or polyps 2 . The American Institute of Radiologic Pathology categorizes lesions <5 mm as “Brunner’s gland hyperplasia” and those >5 mm as “BGH” 3 . The exact pathogenesis of BGH remains unknown. Although it is commonly regarded as a benign duodenal condition, it can enlarge and cause gastrointestinal bleeding or obstruction. In addition, malignant transformation can occur, especially when there is a significant increase in size or the presence of a shallow central depression on its surface 3 . Treatment depends on the tumor size, symptoms, and the possibility of malignancy. Previously, surgical resection was the primary treatment modality. Given however that the BGH is located in the deep mucosa and submucosa, endoscopic resection can be successfully performed, as in the present case, even when it is large.

Endoscopy_UCTN_Code_CCL_1AB_2AC_3AB

## Notes

Endoscopy E-Videos https://eref.thieme.de/e-videos
*E-Videos* is an open access online section of the journal *Endoscopy* , reporting on interesting cases and new techniques in gastroenterological endoscopy. All papers include a high-quality video and are published with a Creative Commons CC-BY license. Endoscopy E-Videos qualify for HINARI discounts and waivers and eligibility is automatically checked during the submission process. We grant 100% waivers to articles whose corresponding authors are based in Group A countries and 50% waivers to those who are based in Group B countries as classified by Research4Life (see: https://www.research4life.org/access/eligibility/ ). This section has its own submission website at https://mc.manuscriptcentral.com/e-videos .
